# *Helicobacter pylori* infection is associated with elevated galactose-deficient IgA1 in IgA nephropathy

**DOI:** 10.1080/0886022X.2020.1772295

**Published:** 2020-06-11

**Authors:** Xing-Zi Liu, Yue-Miao Zhang, Ni-Ya Jia, Hong Zhang

**Affiliations:** a Renal Division, Peking University First Hospital, Peking University Institute of Nephrology, Beijing, People’s Republic of China; b Key Laboratory of Renal Disease, Ministry of Health of China, Key Laboratory of Chronic Kidney Disease Prevention and Treatment (Peking University), Ministry of Education, Beijing, People’s Republic of China

**Keywords:** Immunoglobulin A nephropathy, *Helicobacter pylori*, cytotoxin-associated gene A/vacuolating cytotoxin A, galactose-deficient IgA1

## Abstract

**Background:**

Mucosal immunity plays an important role in the pathogenesis of IgA nephropathy (IgAN). This study aimed to investigate if infection of *Helicobacter pylori* (*H. pylori*), a common bacteria in the gastrointestinal tract, associated with IgAN.

**Methods:**

This study included 261 patients with IgAN and 46 healthy controls. Clinical information and plasma samples were collected from patients and healthy controls. *H. pylori* infection was confirmed by western blot. Plasma IgA1 and galactose-deficient IgA1 (Gd-IgA1) levels were detected by specific enzyme-linked immunosorbent assay.

**Results:**

Total *H. pylori* infection rates showed no statistical differences between IgAN patients and healthy controls, but the infection rates of type I *H. pylori* in IgAN patients were significantly higher than those in healthy controls (44.4 vs. 28.3%, *p* = 0.040). Compared with uninfected patients, the systolic blood pressure, 24-h proteinuria, and blood urea nitrogen levels were significantly higher in patients with *H. pylori* infection (126.0 ± 15.5 vs. 119.6 ± 14.5 mmHg, *p* = 0.010; 1.8 ± 2.7 vs. 1.2 ± 1.4 g/24h, *p* = 0.013; 7.9 ± 5.4 vs. 6.7 ± 3.9 μmol/L, *p* = 0.042), especially in patients with type I infection (126.5 ± 15.4 vs. 119.6 ± 14.5 mmHg, *p* = 0.002; 1.9 ± 2.9 vs. 1.2 ± 1.4 g/24 h, *p* = 0.033; 8.1 ± 5.6 vs. 6.7 ± 3.9 μmol/L, *p* = 0.041). Similarly, patients with IgAN and type I *H. pylori* infection showed higher plasma Gd-IgA1 levels than uninfected patients (5.5 ± 2.2 vs. 4.5 ± 2.2 μg/mL, *p* = 0.037).

**Conclusions:**

Virulent type I *H. pylori* infection is more common in patients with IgAN. Patients with IgAN and type I *H. pylori* infection showed lower renal function and higher underglycosylation of plasma IgA1.

## Introduction

Immunoglobulin A nephropathy (IgAN), the most common cause of glomerulonephritis worldwide, is characterized by the predominant or codominant deposition of IgA in the glomerular mesangium [1]. Increasing evidences suggested that mucosal immunity played an important role in the pathogenesis of IgAN [2,3], and *Helicobacter pylori* (*H. pylori*) infection might be the most common factor [4,5]; however, the associations between *H. pylori* infection and clinical manifestations of IgAN and its possible mechanism have not been elucidated yet.

*H. pylori*, a gram-negative bacterium, colonizes the human gastric mucus layer. Recently, considerable studies changed the paradigm that *H. pylori* only participates in the pathogenesis of chronic gastritis and peptic ulcer disease, and demonstrated that several extra-intestinal diseases, such as renal-related diseases, were also caused by *H. pylori* [6–8]. The systemic antibody response to *H. pylori* was reported to be increased in patients with IgAN [9], suggesting that *H. pylori* might be involved in the pathogenesis and progression of IgAN. Cytotoxin-associated gene A (CagA) and vacuolating cytotoxin A (VacA) are the main virulence factors of *H. pylori* [10]. CagA has been observed in the tonsils of most *H. pylori* infected IgAN patients [11], and it was reported to promote glomerular mesangial cell proliferation and extracellular matrix secretion *via* suppressing the apoptotic signaling pathway [12]. VacA was found to play some roles in vacuolation, apoptosis, antigen presentation, and multiple cellular activities [13,14]. However, the association of the virulent *H. pylori* strains and IgAN clinical manifestations and possible mechanism remained to be investigated.

In this study, we investigated the infection rates of different *H. pylori* types, particularly virulent strains, and the correlation between *H. pylori* infection and clinical manifestations in patients with IgAN. Moreover, the levels of plasma IgA1 and galactose-deficient IgA1 (Gd-IgA1) in patients with different *H. pylori* type infection were detected to explore the possible mechanism.

## Materials and methods

### Study population

A total of 261 patients with IgAN (mean age: 37.7 ± 12.3 years, male ratio: 51.7%) were enrolled in this study from Peking University First Hospital. Forty-six age- and sex-matched healthy individuals (mean age: 38.0 ± 12.2 years, male ratio: 47.8%) were recruited as healthy controls. The patients with IgAN were diagnosed by renal biopsy and confirmed by the deposition of IgA in the glomerular mesangium using immunofluorescence and ultrastructural examination. Patients with secondary causes of IgAN, such as IgA vasculitis, systemic lupus erythematosus, or liver cirrhosis were excluded.

Plasma samples were collected from patients (at renal biopsy) and healthy controls and stored at −80 °C for further use. Baseline demographic and clinical data were collected at the time of renal biopsy, including age, sex, systolic blood pressure (SBP), diastolic blood pressure (DBP), gross/microscopic hematuria, serum creatinine (Scr), serum IgA/IgG/IgM, serum complement 3 (C3), 24-h proteinuria, blood urea nitrogen (BUN), treatment regimes, and histological characteristics. Estimated glomerular filtration rate (eGFR) was calculated using the Chronic Kidney Disease Epidemiology Collaboration equation [15]. Twenty-four hour creatinine clearance (CrCl) was done by the Cockcroft–Gault formula [16]. The histological characteristics were scored according to the Oxford classification [17].

### Detection of *H. pylori* infection

Serum *H. pylori-*IgG antibodies (CagA, VacA, UreA, and UreB) were detected in all participants using the Typing Detection Kit (Blot Biotech, Shenzhen, China), according to the manufacturer’s protocol. Seropositivity for UreA and/or UreB IgG confirmed *H. pylori* infection. Type I *H. pylori* infection was diagnosed if seropositivity for CagA and/or VacA IgG, otherwise, the type II *H. pylori* infection was diagnosed. The diagnosis of *H. pylori* infection is summarized in Supplementary Table 1.

### Detection of plasma IgA1 and galactose-deficient IgA1

The levels of plasma IgA1 in patients with IgAN were detected by enzyme-linked immunosorbent assay (ELISA), according to a previously described protocol [18]. Briefly, 96-well plates were coated with F(ab’)_2_ fragments of goat IgG anti-human IgA overnight at 4 °C, followed by blocking with 1% bovine serum albumin for 1 h at 37 °C. Next, plasma samples (1:80,000 dilution) and standard samples were added to the 96-well plates and incubated for 1 h at 37 °C, followed by treatment with horseradish peroxidase-conjugated monoclonal anti-human IgA1 antibodies and tetramethylbenzidine liquid substrate. The absorbance was then measured at 450/570 nm with a microplate reader (Bio-Rad, Japan). Plasma Gd-IgA1 levels were detected using the KM55 ELISA kit (IBL, Japan) [19,20]. Briefly, the ELISA plates were incubated with plasma samples (1:400 dilution in EIA buffer) and standard samples for 1 h at 37 °C, washed four times with wash buffer, incubated with prepared-labeled antibodies, and then treated with 50 µL TMA solution for 30 min in the dark. The absorbance was measured at 450/630 nm by an ELISA reader (Bio-Rad, Japan). The reference range of IgA1 and Gd-IgA1 levels was calculated according to their respective standard curves generated from parallel working standards.

### Statistical analysis

Continuous variables with normal distribution were expressed as mean ± standard deviation (SD) and non-normal variables were presented as median and interquartile range (IQR). Categorical variables were reported as absolute frequencies and percentages. For comparison of normally distributed continuous variables, the independent samples *t*-test was used. For analysis of non-normally distributed data, Mann–Whitney *U* test or Kruskal–Wallis test was used. The chi-squared test was performed for comparison of categorical variables. Pearson’s correlation and linear regression analyses were used to determine the association between two continuous variables. All results were analyzed by SPSS version 22.0 (SPSS Inc., Chicago, USA) and expressed as hazard ratios with 95% confidence intervals. A two-tailed P value less than 0.05 was considered statistically significant.

## Results

### The baseline characteristics of patients with IgAN

Two hundred and sixty-one patients with IgAN were enrolled in this study. The baseline characteristics, including age, sex, SBP, DBP, gross/microscopic hematuria, Scr, serum IgA/IgG/IgM, serum C3, 24-h proteinuria, eGFR, CrCl, BUN, treatment regimes, and histological characteristics, were demonstrated in Supplementary Table 2.

### *H. pylori* infection rates

**Figure 1.**
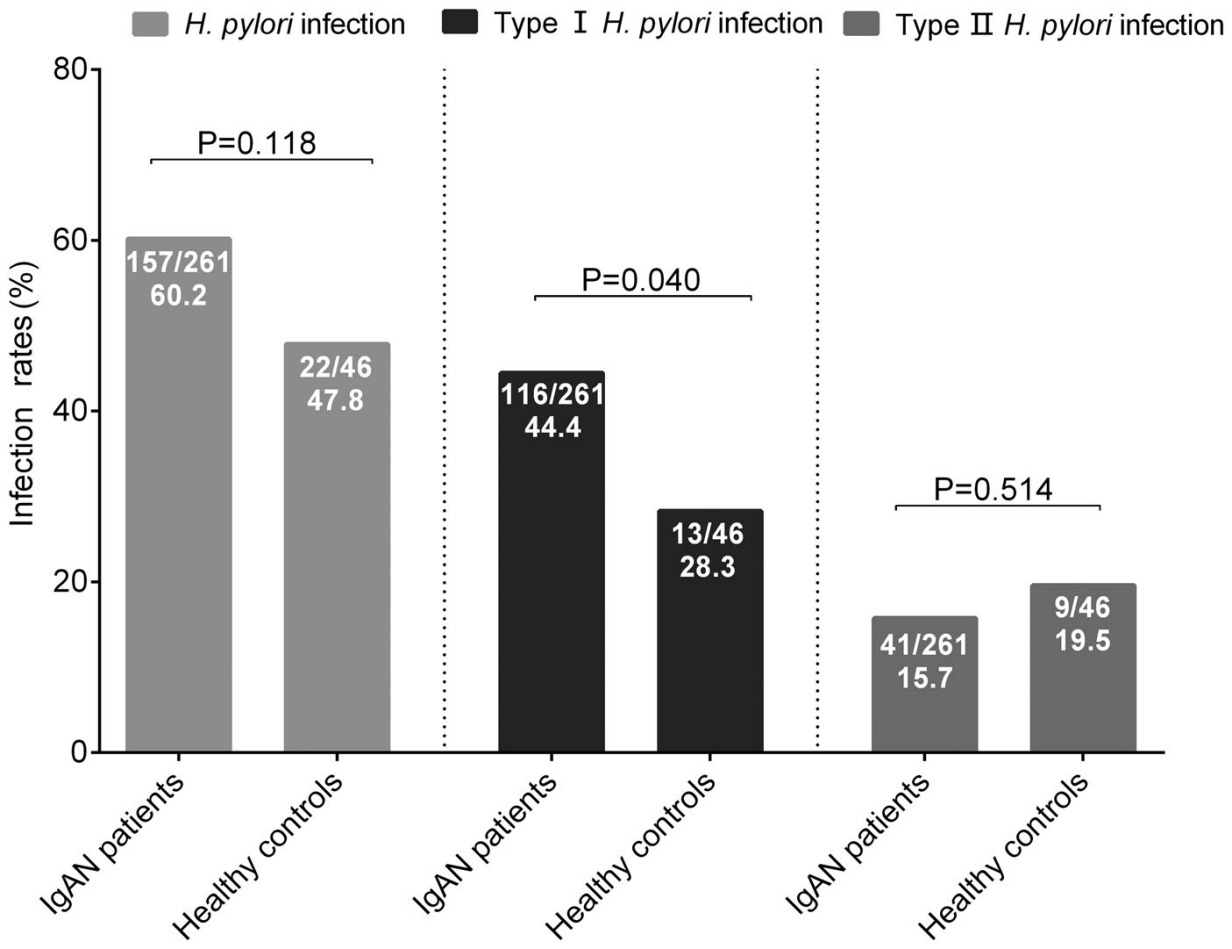
The *Helicobacter pylori* infection rates in IgA nephropathy patients and healthy controls. IgAN: IgA nephropathy; *H. pylori*: *Helicobacter pylori*.

*H. pylori* infection rates in patients with IgAN tended to be higher than those in healthy controls (157/261, 60.2% vs. 22/46, 47.8%; *p* = 0.118). Subgroup analyses showed that there were significant higher type I *H. pylori* infection rates in patients with IgAN, as compared with healthy controls (116/261, 44.4% vs. 13/46, 28.3%; *p* = 0.040). While, type II *H. pylori* infection rates were comparable between IgAN patients and healthy controls (41/261, 15.7% vs. 9/46, 19.5%, *p* = 0.514) (Figure 1).

### Association between *H. pylori* infection and clinical manifestations

Compared with uninfected IgAN patients, patients with *H. pylori* infection showed significantly higher SBP (126.0 ± 15.5 vs. 119.6 ± 14.5 mmHg; *p* = 0.010), 24-h proteinuria (1.8 ± 2.7 vs. 1.2 ± 1.4 g/24h; *p* = 0.013), and BUN (7.9 ± 5.4 vs. 6.7 ± 3.9 μmol/L, *p* = 0.042) levels. Further subgroup analyses showed that the above indicators were significantly higher in IgAN patients with type I *H. pylori* infection (126.5 ± 15.4 vs. 119.6 ± 14.5 mmHg, *p* = 0.002; 1.9 ± 2.9 vs. 1.2 ± 1.4 g/24h, *p* = 0.033; 8.1 ± 5.6 vs. 6.7 ± 3.9 μmol/L, *p* = 0.041), but not in IgAN patients with type II *H. pylori* infection (124.5 ± 16.0 vs. 119.6 ± 14.5 mmHg, *p* = 0.081; 1.8 ± 2.0 vs. 1.2 ± 1.4 g/24h, *p* = 0.088; 7.7 ± 4.7 vs. 6.7 ± 3.9 μmol/L, *p* = 0.211). And no significant differences were observed in age, sex, SBP, DBP, gross/microscopic hematuria, Scr, serum IgA/IgG/IgM, serum C3, 24-h proteinuria, eGFR, CrCl, BUN, treatment regimes, and histological characteristics between patients with type I infection and patients with type II infection (Table 1).

**Table 1.** Association of *H. pylori* infection and clinical manifestation in patients with IgA nephropathy.

	Uninfected (*n* = 104)	*H. pylori* infection (*n* = 157)	Type I *H. pylori* infection (*n* = 116)	Type II *H. pylori* infection (*n* = 41)	*p*^a^ value	*p*^b^ value	*p*^c^ value	*p*^d^ value
Age, years	37.1 ± 11.9	38.1 ± 12.5	39.0 ± 13.4	35.6 ± 9.3	0.500	0.258	0.473	0.075
Sex, male, *n* (%)	50 (48.1)	85 (54.1)	61 (52.6)	24 (58.5)	0.337	0.504	0.257	0.511
SBP, mmHg	119.6 ± 14.5	126.0 ± 15.5	126.5 ± 15.4	124.5 ± 16.0	0.010	0.002	0.081	0.101
DBP, mmHg	75.1 ± 11.3	77.3 ± 10.5	77.2 ± 9.8	77.5 ± 12.4	0.109	0.138	0.257	0.593
Hypertension, *n* (%)	42 (40.4)	73 (46.5)	59 (50.9)	14 (34.1)	0.330	0.119	0.487	0.065
Gross hematuria, *n* (%)	26 (25.0)	37 (23.6)	28 (24.1)	9 (22.0)	0.791	0.882	0.699	0.777
Microscopic hematuria, *n* (%)	87 (83.7)	137 (87.3)	100 (86.2)	37 (90.2)	0.246	0.399	0.221	0.505
Serum IgG, g/L	10.8 ± 3.2	10.9 ± 3.1	11.0 ± 3.3	10.1 ± 2.8	0.916	0.909	0.248	0.397
Serum IgA, g/L	3.3 ± 1.2	3.4 ± 1.1	3.3 ± 1.1	3.4 ± 1.2	0.510	0.674	0.930	0.989
Serum IgM, g/L	1.3 ± 0.7	1.4 ± 3.3	1.5 ± 3.8	1.1 ± 0.6	0.671	0.464	0.231	0.550
Serum C3, g/L	0.9 ± 0.2	0.9 ± 0.2	0.9 ± 0.2	0.9 ± 0.2	0.672	0.736	0.800	0.879
Proteinuria, g/24 h	1.2 ± 1.4	1.8 ± 2.7	1.9 ± 2.9	1.8 ± 2.0	0.013	0.033	0.088	0.592
Scr, μmol/L	126.4 ± 116.0	149.8 ± 144.4	148.5 ± 128.9	153.5 ± 183.0	0.171	0.187	0.292	0.851
eGFR, mL/min/1.73m^2^	77.8 ± 36.7	72.3 ± 37.0	71.8 ± 37.8	73.7 ± 34.9	0.240	0.236	0.546	0.803
CrCl, mL/min	82.8 ± 43.4	77.1 ± 40.4	76.9 ± 42.4	77.4 ± 34.9	0.284	0.322	0.477	0.952
BUN, μmol/L	6.7 ± 3.9	7.9 ± 5.4	8.1 ± 5.6	7.7 ± 4.7	0.042	0.041	0.211	0.687
Treatment regimes, *n* (%)								
ACE inhibitors or ARBs	100 (96.2)	153 (97.5)	114 (98.3)	39 (95.1)	0.551	0.335	0.779	0.271
Immunosuppressive agents	34 (32.7)	48 (30.6)	36 (31.3)	12 (29.3)	0.718	0.792	0.690	0.833
Prednisone	*39 (37.5)*	*52 (33.1)*	42 (36.6)	10 (24.4)	0.467	0.843	0.133	0.167
Oxford Score, *n* (%)								
M0/1	*30/74 (28.8/71.2)*	*53/104 (33.8/66.2)*	41/75 (35.3/64.7)	12/29 (29.3/70.7)	0.404	0.303	0.960	0.479
E0/1	*66/38 (63.5/36.5)*	*104/53 (66.2/33.8)*	75/41 (64.7/35.3)	29/12 (70.7/29.3)	0.644	0.854	0.407	0.479
S0/1	*31/73 (29.8/70.2)*	*49/108 (31.2/68.8)*	40/76 (34.5/65.5)	9/32 (22.0/78.0)	0.810	0.459	0.340	0.132
T0/1/2	*54/41/9 (49.0/39.4/11.6)*	*72/72/13 (45.9/45.9/8.2)*	50/55/11 (43.1/47.4/9.5)	22/17/2 (53.7/41.5/4.8)	0.581	0.418	0.741	0.418
C0/1/2	86/13/5 (82.7/12.5/4.8)	130/20/7 (82.8/12.7/4.5)	96/16/7 (80.2/13.8/6.0)	37/4/0 (90.2/9.8/0.0)	0.990	0.876	0.308	0.214

ACE: angiotensin-converting enzyme; ARB: angiotensin II receptor blocker; BUN: blood urea nitrogen; C: crescents; CrCl: 24-h creatinine clearance; DBP: diastolic blood pressure; E: Endocapillary proliferation; eGFR: estimated glomerular filtration rate; SBP: systolic blood pressure; Scr: serum creatinine; M: mesangial hypercellularity; S: segmental sclerosis; T: interstitial fibrosis and tubular atrophy.

*p*^a^ value: *H. pylori* infection group versus uninfected group; *p*^b^ value: type I *H. pylori* infection group versus uninfected group; *p*^c^ value: type II *H. pylori* infection group versus uninfected group; *p*^d^ value: type I *H. pylori* infection group versus type II *H. pylori* infection group.

### Plasma IgA1 and galactose-deficient IgA1 levels

**Figure 2.**
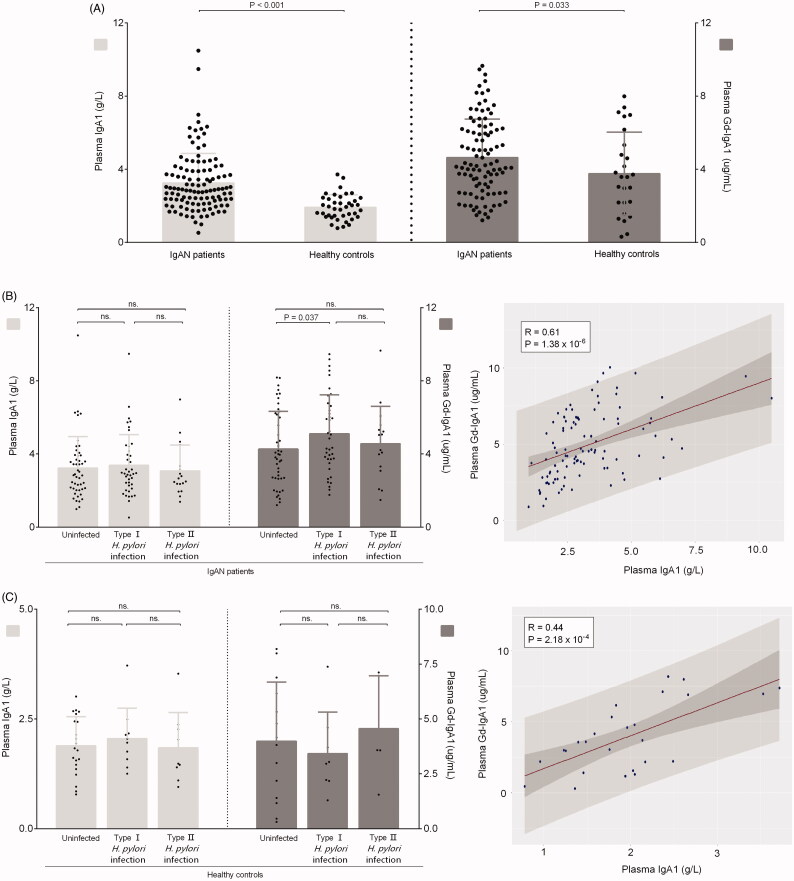
Levels of plasma IgA1 and galactose-deficient IgA1 in participants. (A) The plasma IgA1 and Gd-IgA1 levels in patients with IgAN and healthy controls. (B) The Plasma IgA1 and Gd-IgA1 levels in IgAN patients with or without *H. pylori* infection. (C) The Plasma IgA1 and Gd-IgA1 levels in healthy controls with or without *H. pylori* infection.

Plasma IgA1 and Gd-IgA1 levels were both significantly higher in patients with IgAN than those in healthy controls (3.3 ± 1.6 vs. 2.1 ± 1.0 g/L; *p* < 0.001; 5.0 ± 2.2 vs. 3.9 ± 2.4 μg/mL, *p* = 0.033), which were the common disease characteristics (Figure 2(A)). Plasma Gd-IgA1 levels were significantly higher in patients with type I infection than in uninfected patients (5.5 ± 2.2 vs. 4.5 ± 2.2 μg/mL, *p* = 0.037). But there was no significance between patients with type I and type II *H.pylori* infection (5.5 ± 2.2 vs. 4.9 ± 1.9 μg/mL, *p* = 0.344). Conversely, compared with uninfected patients, plasma IgA1 levels were comparable with patients with type I infection (3.2 ± 1.7 vs. 3.4 ± 1.7 g/L, *p* = 0.693) and patients with type II *H. pylori* infection (3.2 ± 1.7 vs. 3.1 ± 1.4 g/L, *p* = 0.723). Further correlation analyses showed a significantly positive correlation between plasma IgA1 and Gd-IgA1 levels (correlation coefficient = 0.61, *p* = 1.38 × 10^−6^) in patients with IgAN (Figure 2(B)). While, in controls, there were no significant differences both in plasma IgA1 and Gd-IgA1 levels whether between uninfected controls and controls with type I infection (1.9 ± 0.7 vs. 2.1 ± 0.7 g/L, *p* = 0.535; 4.0 ± 2.7 vs. 3.4 ± 1.9 μg/mL, *p* = 0.608) or between controls and controls with type II infection (1.9 ± 0.7 vs. 1.8 ± 0.8 g/L, *p* = 0.869; 4.0 ± 2.7 vs. 4.6 ± 2.4 μg/mL, *p* = 0.682) or between controls with I infection and controls with II infection (2.1 ± 0.7 vs. 1.8 ± 0.8 g/L, *p* = 0.552; 3.4 ± 1.9 vs. 4.6 ± 2.4 μg/mL, *p* = 0.363) (Figure 2(C)).

## Discussion

In this study, patients with IgAN showed higher infection rates of *H. pylori*, especially type I *H. pylori*, than healthy controls. And IgAN patients infected with *H. pylori*, especially type I *H. pylori*, showed significantly higher SBP, 24-h proteinuria, and BUN levels than uninfected patients. Similarly, higher plasma Gd-IgA1 levels were observed in IgAN patients with type I *H. pylori* infection.

Mucosal infection was reported to be involved in the development of IgAN through triggering innate and adaptive immune responses and the alternative complement pathway [21,22]. Recently, *H. pylori* infection and its linkage with extra-gastric diseases has been widely studied [23,24]. However, whether the infection rates of *H. pylori* are higher in patients with IgAN remains controversial [5,9]. In this study, we recruited 261 patients with IgAN and 46 healthy controls, and results showed that there were slight differences in the infection rates of *H. pylori* between these two groups, but not reach statistical significance; however, the infection rates of type I *H. pylori*, the main virulent strain of *H. pylori*, were significantly higher in patients with IgAN. *H. pylori* was reported to increase proteinuria and renal vasoconstriction in several renal disease as an infection factor through altering the permeability of the glomerular basement membrane, damaging endothelial dysfunction, and promoting production of inflammatory cytokines [25,26], suggesting that *H. pylori* was involved in progression of renal disease. Previous study [27] and our clinical practice (data not included) showed that *H. pylori* eradication reduced proteinuria in patients with IgAN. Decrease trend of eGFR levels were observed in IgAN patients with *H. pylori* infection [4], but the association between different types of *H. pylori* infection and clinical manifestations of IgAN remained to be elucidated. In this study, a trend of lower eGFR was showed in patients with type I infection, but it does not reach statistical significance. And significantly higher 24-h proteinuria, SBP, and BUN were showed in IgAN patients with type I infection, but not in patients with type II infection, implying that IgAN patients with virulent *H. pylori* infection may have lower renal function.

IgA1 with high lectin binding was produced in response to mucosal *H. pylori* infection [28], and exaggerated systemic antibody response, mainly serum anti-*H. pylori* IgA1, to mucosal infection was demonstrated in IgAN patients with *H. pylori* infection [9]. CagA, a crucial virulence factor of type I *H. pylori*, was reported to promote the production and undergalactosylation of IgA1 in the B cell line, DAKIKI [9,29]. It was reported that aberrant glycosylation of IgA1 was involved in the pathogenesis of IgAN [30]. However, the effects of different *H. pylori* strains on plasma Gd-IgA1 levels of IgAN patients remain unclear. In present study, patients with IgAN displayed significantly higher plasma IgA1 and Gd-IgA1 levels than controls, which are the common disease characteristics. And the plasma Gd-IgA1 levels were significantly higher in patients with type I *H. pylori* infection than those in uninfected patients, while the levels in controls and controls with *H. pylori* infection were comparable, indicating that the higher plasma Gd-IgA1 observed in IgAN patients with virulent *H. pylori* infection was associated with the disease, and not a general change in healthy controls. This was consistent with previous studies that the levels of serum mucosal-type IgA1 against *H. pylori* were significantly higher in IgAN patients than healthy controls [9] and the degree of *H. pylori* antigen and CagA deposition were obviously severe in IgAN patients than patients with non-IgAN primary glomerulonephritis [4]. The immune response to *H. pylori* infection may be more stronger in IgAN patients than controls.

The main limitations in our study were as followed. We evaluated the serum anti-*H. pylori* IgG to identify individuals exposed to *H. pylori* infection rather than testing the current infection status using ^13^C-UBT. However, the seropositivity of anti-*H. pylori* IgG could not be converted to seronegativity without *H. pylori* eradication therapy. To a certain extent, the serum anti-*H. pylori* IgG represents current infection and has been used for several epidemiological investigations [31, 32]. Although Gd-IgA1 was suggested to play a central role in pathogenesis of IgAN. Some studies reported that Gd-IgA1 was unrelated with proteinuria in IgAN patients [33,34]. While others observed a trend of lower eGFR, higher proteinuria, and increased use of immunosuppressives in IgAN patients with higher Gd-IgA1 levels [18]. In this observation study, we found that the IgAN patients with type I *H. pylori* infection showed lower renal function and higher plasma Gd-IgA1. Further cohort and *H. pylori* eradication studies are needed to verify whether *H. pylori* infection participate in the pathogenesis and progression of IgAN through the elevated plasma Gd-IgA1.

## Conclusions

In conclusion, our study revealed that virulent type I *H. pylori* infection was more common in patients with IgAN. And *H. pylori* infection, especially type I *H. pylori* infection, was associated with higher 24-h proteinuria, SBP, and BUN, as well as higher plasma Gd-IgA1 levels in patients with IgAN.

## Supplementary Material

Supplemental Material
