# Supplementary material for: Helicobacter pylori infection is associated with elevated galactose-deficient IgA1 in IgA nephropathy
Source: Ren Fail. 2020 Jun 11;42(1):539–46. doi: 10.1080/0886022X.2020.1772295 (PMC7946026; doi:10.1080/0886022X.2020.1772295)
Supplement: Supplemental Material [file IRNF_A_1772295_SM2601.pdf]

Supplementary Table 1. Principles of *Helicobacter pylori* infection diagnosis.

Types of *H. pylori* infection	
Uninfected	Seronegative for UreA and UreB IgG
Type [illegible] *H. pylori* infection	Seropositive for UreA and/or UreB IgG + CagA and/or VacA IgG
Type [illegible] *H. pylori* infection	Seropositive for UreA and/or UreB IgG + seronegative for CagA and VacA IgG

UreA/B: urease A/B; CagA: cytotoxin associated A; VacA: vacuolating cytotoxin A.

Supplementary Table 2. Baseline characteristics of patients with IgA nephropathy.

	IgAN patients (n=261)
Age, years	37.7±12.3
Sex, male, *n* (%)	135 (51.7)
SBP, mmHg	123.5±15.4
DBP, mmHg	76.4±10.8
Hypertension, *n* (%)	115 (44.1)
Gross hematuria, *n* (%)	63 (24.1)
Microscopic hematuria, *n* (%)	224 (85.8)
Serum IgG, g/L	10.8±3.2
Serum IgA, g/L	3.3±1.1
Serum IgM, g/L	1.4±2.6
Serum C3, g/L	0.9±0.2
Proteinuria, g/24 h	1.6±2.3
Scr, µmol/L	140.6±134.2
eGFR, mL/min/1.73m^2	74.4±36.9
CrCl, mL/min	79.3±41.6
BUN, µmol/L	7.5±4.9
Treatment regimes, *n* (%)	
ACE inhibitors or ARBs	253 (96.9)
Immunosuppressive agents	82 (31.4)
Prednisone	91 (34.9)
Oxford Score, *n* (%)	
M0/1	83/178 (31.8/68.2)
E0/1	170/91 (65.1/34.9)
S0/1	80/181 (30.7/69.3)
T0/1/2	126/113/22 (48.3/43.3/8.4)
C0/1/2	216/33/12 (82.8/12.6/4.6)

IgAN: IgA nephropathy; SBP: systolic blood pressure; DBP: diastolic blood pressure; Scr: serum creatinine; eGFR: estimated glomerular filtration rate; CrCl: 24-hour creatinine clearance; BUN: blood urea nitrogen; ACE: angiotensin-converting enzyme; ARB: angiotensin II receptor blocker; M: Mesangial hypercellularity; E: Endocapillary proliferation; S: Segmental sclerosis; T: interstitial fibrosis and tubular atrophy; C: Crescents.
